# Influential Factors of Masticatory Performance in Older Adults: A Cross-Sectional Study

**DOI:** 10.3390/ijerph18084286

**Published:** 2021-04-18

**Authors:** Hee-Eun Kim

**Affiliations:** Department of Dental Hygiene, Gachon University College of Health Science, Incheon 21936, Korea; hekim@gachon.ac.kr; Tel.: +82-32-820-4375

**Keywords:** chewing ability, functional tooth unit, masticatory performance, masseter muscle, mixing ability index

## Abstract

While appropriate nutrient intake is important for older adults, various oral problems cause decreased masticatory function. This cross-sectional study aimed to identify the factors associated with decreased masticatory performance in older adults. Oral examinations were performed on 80 participants (mean age: 75.10 ± 5.64 years) to determine the number of functional tooth units (FTUs). Symptoms of periodontal and temporomandibular diseases were evaluated via a questionnaire. The tone, elasticity, and dynamic stiffness of the masseter muscle were measured using the Myoton^®^ PRO device. The mixing ability test was used to assess the masticatory performance, and the mixing ability index (MAI) was calculated. The analysis of covariance test was performed to adjust for confounding factors, and multiple logistic regression analysis was performed to identify the risk factors affecting MAI. A lower MAI was significantly associated with higher tone (*p* = 0.006) and lower elasticity (*p* = 0.013). The number of FTUs (adjusted odds ratio (OR) = 0.724, *p* = 0.029), tone (adjusted OR = 1.215, *p* = 0.016), and elasticity (adjusted OR = 4.789, *p* = 0.038) were independently associated with the MAI. Muscle function training and prosthetic treatments may help increase masticatory performance in older adults, which would improve overall health.

## 1. Introduction

With the advent of a super-aged society, interest in healthy aging is increasing. Successful health management of older adults begins with a balanced intake of nutrients [1]. However, this population experiences various oral problems, including tooth loss, leading to decreased masticatory function, which subsequently leads to nutritional imbalances accompanied by systemic diseases [2].

There are numerous factors that affect masticatory function. Previous studies have shown that decreased number of remaining teeth, occlusal force, salivary secretion rate, and pain in the oral and maxillofacial region in the elderly are associated with decreased chewing ability [3,4]. Additionally, neuropsychiatric symptoms, such as decreased nerve activity and circulation in the oral and maxillofacial region, cognitive decline, depression, and dementia, have been associated with chewing ability [5,6,7]. The most significant factor affecting chewing ability is the number and distribution of remaining teeth [8].

However, mastication is a complex motion achieved by not only the teeth but also the temporomandibular joints and masticatory muscles [9]. Tooth loss, temporomandibular joint pain, and reduced masticatory muscle function due to aging may decrease masticatory function [4,10,11]. However, most studies have only compared the relationship between masticatory function and risk factors, and none have comprehensively analyzed the effects of risk factors on masticatory function while controlling for possible confounding variables. Particularly, there is a clear lack of objective data to support the critical relationship between masticatory function and masticatory muscle function. Higashi [12] reported that activities of the masticatory muscles affect the chewing movement; however, only the changes in the state of the masticatory muscles during opening and closing of the oral cavity were evaluated fragmentarily, and masticatory function was not measured. Ohara et al. [13] showed that the strength of the masseter muscle tension on palpation was significantly different depending on masticatory function; however, this study had conducted a subjective assessment using a self-reported questionnaire, and the bite force was measured in a static state to evaluate masticatory function. As mastication is a series of motions, including biting, cutting, chewing, and crushing, the static occlusal force does not fully reflect the complex and dynamic masticatory motions of the oral and maxillofacial system [14]. A comprehensive understanding of the effects of the teeth, temporomandibular joints, and masticatory muscles on masticatory function is necessary [14].

Masticatory function can be evaluated using an objective assessment of masticatory performance and a subjective evaluation of masticatory ability. The mixing ability test is the standard method of evaluation of masticatory performance [15]. In this test, a two-color wax is masticated, and the ratio of color mixing is analyzed to calculate the mixing ability index (MAI), which reflects the dynamic processes of chewing in the oral cavity. Sato et al. [16] reported that the mixing ability test has a higher validity than the conventional sieving method in evaluating the masticatory function. Ikebe et al. [17] also stated that the mixing ability test is a valid method for evaluating masticatory performance. In addition, Park et al. [18] demonstrated that masticatory function measured using the MAI was significantly decreased at 4 and 8 weeks after injection of botulinum toxin type A into the masseter muscles. In contrast, subjective assessment of masticatory ability is conducted using the food intake questionnaire, which evaluates the food intake ability (FIA) of participants via a questionnaire to determine their ability to chew foods of varying hardness. Sakurai et al. [19] evaluated the masticatory ability for 31 foods via the FIA and reported that the FIA is useful in oral health education and clinical practice; moreover, Koshino et al. [20] reported that a food intake questionnaire consisting of 25 food items was valid and reliable in evaluating masticatory ability. However, in older adults, the mixing ability test is believed to be a more accurate method than other conventional methods of assessing masticatory function [21].

Therefore, it may be stated that appropriate nutritional intake and normal masticatory function are essential to promote healthy aging. When masticatory function is impaired in older adults, dental treatments are primarily considered. However, these are different from community-based integrated oral health management strategies. Consequently, a comprehensive analysis of the factors that affect masticatory function in community-dwelling older adults is necessary for establishing additional management strategies that effectively improve masticatory function. Therefore, this study aimed to analyze the relationship between masticatory performance and the factors affecting the functional occlusion system and to identify the risk factors responsible for the decline in masticatory performance.

## 2. Materials and Methods

### 2.1. Ethical Aspects

This cross-sectional study was approved by the Institutional Review Board of Gachon University (IRB No. 1044396-201612-HR-105-01). All procedures of this study were conducted in accordance with the ethical principles for medical research involving human participants as stipulated in the Declaration of Helsinki (2013 version) by the World Medical Association and the Strengthening the Reporting of Observational Studies in Epidemiology (STROBE) guidelines [22]. The purpose and methods of the study were explained in detail to all the participants, and written informed consent was obtained from them.

### 2.2. Participants

The sample size was calculated by applying the correlation analysis model of G*power 3.1 software (Heinrich-Heine-University Düsseldorf, Düsseldorf, Germany). Based on the results of the study by Morita et al. [23] and those of a preliminary study, a total of 79 participants were required, with a correlation coefficient of *r* = 0.152, 95% power, and an alpha level of 0.05. A 10% drop-out rate was taken into account, resulting in the recruitment of 87 participants. The participants of this study were selected through convenience sampling. Among the elderly individuals aged ≥65 years living in Yeonsu-gu, Incheon, 87 who visited the institute were consecutively recruited from July 2017 to September 2018. These participants did not have any infectious diseases or uncontrolled systemic diseases, could move independently, and had voluntarily expressed their intention to participate. The final sample consisted of 80 elderly individuals selected based on the inclusion and exclusion criteria. After an interview to confirm medical and dental history, three edentulous patients and four patients with prosthetic treatment plans were excluded. The inclusion criterion was the presence of permanent dentition (except the third molars). The exclusion criteria were factors that could compromise masticatory evaluation, such as painful dental caries, community periodontal index of 4 points or higher, orofacial pain, complete dentures, planned prosthetic treatment, and poor literacy.

### 2.3. Oral Examination

Oral examinations were performed by a single trained dental hygienist, calibrated with an intraclass correlation coefficient of 0.82. The participants’ dentures, number of remaining teeth, and number of functional tooth units (FTUs) were assessed. Remaining teeth referred to all teeth capable of mastication, except for the third molars, including sound teeth, filled teeth, and teeth with enamel or dentin caries, as well as teeth with fixed prosthesis [24]. FTUs included pairs of opposing teeth of the upper and lower jaws, except for the third molar, and the numbers of FTUs for the molar and premolar teeth were 2 and 1, respectively [25]. Only premolars and molars were included as functional teeth as they are primarily involved in mastication. If there were no missing teeth, the number of FTUs was 12 [26]. There were six conditions of paired teeth (natural tooth-natural tooth, fixed prosthesis-natural tooth, fixed prosthesis-fixed prosthesis, removable prosthesis-natural tooth, removable prosthesis-fixed prosthesis, removable prosthesis-removable prosthesis) [25], and the FTUs for removable prosthesis pairs were calculated as follows, considering a functional recovery rate of 50%: FTUs × 0.5 [26]. Questions regarding the subjective symptoms of bleeding and swelling of the gums as well as teeth extrusion and mobility in the 3 months before the study were used to evaluate the periodontal status of the participants. A score of 1 was assigned for each of the subjective symptoms, while a score of 0 was assigned for no subjective symptoms. Therefore, the score for symptoms related to periodontal disease ranged from a minimum score of 0 to a maximum score of 4 (see Supplementary Materials, File S1, for the questionnaire). Additionally, self-reported symptoms related to temporomandibular disease (TMD) were also evaluated. Pain, clicking, and trismus of the temporomandibular joints in the last 3 months before the study were assessed, and scores of 1 and 0 were assigned for the presence and absence of these symptoms, respectively. Therefore, the score for symptoms related to TMD ranged from a minimum score of 0 to a maximum score of 3 (see Supplementary Materials, File S2, for the questionnaire). The salivary secretion rate was assessed as follows: stimulated whole saliva was collected in a 50 mL tube while the participant chewed paraffin wax for 5 min. The value was converted into the salivary secretion rate per minute (mL/min).

### 2.4. Assessment of the Tone and Biomechanical Properties of the Masseter Muscle

The participants were asked to place the lower jaw in the physiological rest position. This lower jaw position was reproduced with a free-way space of 2–3 mm without the upper and lower teeth touching each other. In the centric occlusion position, occlusion was induced with the maximum force, and the area in the masseter muscles that contracted the most were marked as assessment points. The probe of a handheld Myoton^®^ PRO device (MyotonPRO, Myoton Ltd., Tallinn, Estonia) was placed perpendicular to the skin surface overlying the muscles and constant pressure (0.18 N) was applied; the device delivered a force of 0.4 N on the underlying soft tissue for 15 ms to cause muscle deformation. The resultant damped natural oscillations caused by the viscoelastic properties of the biological tissue were recorded using a built-in accelerometer at a sampling rate of 3200 Hz. Under the same conditions, identical points of the left and right masseter muscles were evaluated five times each. The tone and biomechanical properties of masseter muscles were calculated using the MyotonPRO software. The tone of the muscle refers to the inherent vibration of the muscles in the absence of spontaneous muscle contraction; it indicates the intrinsic tension state (natural oscillation frequency, Hz) of the muscle at rest. The higher the oscillation frequency, the greater the muscle tension that increases via contraction [27]. Biomechanical properties, including elasticity and dynamic stiffness, were assessed. The elasticity of the muscle is characterized by the logarithmic decrement of a muscle’s natural oscillation and represents the muscle’s ability to recover its initial shape after deformation. A lower value of decrement reveals smaller dissipation of mechanical energy and better muscle elasticity [27]. Dynamic stiffness of the muscle (Newtons/meter, N/m) is the ability to resist a contraction or an external force that modifies the muscle’s initial shape. The higher the N/m value, the stiffer the muscle [27] and the lesser the muscle relaxation.

### 2.5. Assessment of Masticatory Performance

**Figure 1:**
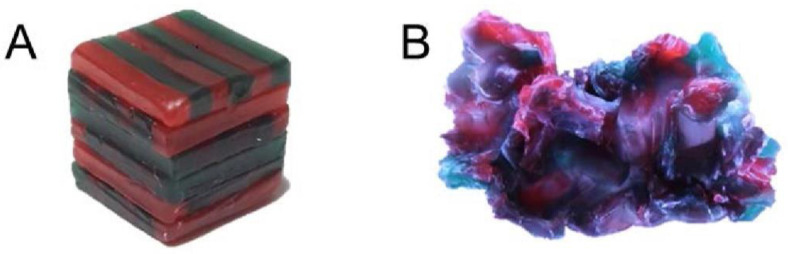
Preparation of a two-color wax cube (**A**) and a chewed wax cube (**B**).

The mixing ability test was performed to assess objective masticatory performance. A wax cube was masticated 10 times, and the total area of the wax specimen, the level of puncture, and the level of color mix were comprehensively evaluated to determine the MAI [15]. The wax cube was made by arranging red and green utility wax rods (Daedong Industrial Co., Ltd., Daegu, Korea) without any overlap in a 12 × 12 × 12 mm cube (Figure 1A) [28]. The participants were asked to masticate one wax cube with their right teeth 10 times, and the procedure was repeated with their left teeth (Figure 1B). The front and back of the masticated specimens were photographed using a digital camera (Canon EOS 500D, Canon Korea Consumer Imaging Inc., Seoul, Korea) at the same distance between the lens and the specimen and under the same light source. The shooting conditions of the camera were as follows: ISO = 100, shutter speed = 1/40, and aperture = 5.6. The specimen images were saved as JPEG files, and an image program (Image-pro 10.0, Media Cybernetics Inc., Silver Spring. MD, USA) was used to analyze the following values: total projection area (TPA, mm^2^), projection area less than 50 μm in thickness (P, mm^2^), maximum length (ML, mm), maximum breadth (MB, mm), red area (RA), and green area (GA). The following variables were calculated using the six analyzed values: the ratio of the area mixed with two colors (MIX) = 100 − (RA + GA)/P × 100, the ratio of the area below 50 μm in thickness to TPA (TR) = 100 − P/TPA × 100, the proportion of maximum length to breadth (LB) = ML/MB, and the shape factor, which showed how flat the sample was (FF) = ML2 × π/4 × TPA × 100. The MIX, TR, LB, and FF were used to calculate the MAI [15,28]. The mean MAI value of the front and back sides of the masticated wax cube was used to calculate the total MAI, and the MAI of each participant was recorded as the mean MAI of the two wax cubes masticated with the right and left teeth. The calculated MAIs were converted to a 0–100-point scale, and a higher MAI score was considered to indicate a higher masticatory performance.

### 2.6. Statistical Analyses

The Shapiro–Wilk test was used to determine the normality of the data (*p* = 0.118), and Pearson’s correlation analysis was performed to identify variables related to the MAI. Analysis of covariance (ANCOVA) was performed using FTUs, symptoms related to periodontal disease, and symptoms related to TMD as covariates to adjust for confounding factors. After classifying the MAI into two groups based on median value, a multiple logistic regression analysis with backward elimination was performed to identify the risk factors affecting the MAI. All statistical analyses were performed using SPSS version 25 statistical software (IBM Chicago, IL, USA). Statistical significance was set at *p* < 0.05.

## 3. Results

Table 1 presents the oral and maxillofacial characteristics that may affect the masticatory function of the 80 participants. A correlation analysis was performed to assess the relationship of these factors with MAI (Table 2). A higher number of remaining teeth (*r* = 0.270, *p* = 0.015) and FTUs (*r* = 0.404, *p* < 0.001) were significantly associated with a higher MAI, whereas lower scores of symptoms related to periodontal disease (*r* = −0.307, *p* = 0.006) and TMD (*r* = −0.283, *p* = 0.011) were significantly associated with a lower MAI. Additionally, higher tension (*r* = −0.357, *p* = 0.001) and stiffness (*r* = −0.371, *p* = 0.001) and lower elasticity (*r* = −0.317, *p* = 0.004) of the masseter muscles were significantly associated with lower MAI.

The state of the masseter muscles, according to the MAI, was compared after adjusting for the number of FTUs and the score of TMD-related symptoms, both of which were found to impact the MAI (Table 3). The tension of the masseter muscles in the high MAI group was significantly lower than that in the low MAI group (difference: 2.32 ± 0.81 Hz; *p* = 0.006). The elasticity of the masseter muscles in the high MAI group was significantly higher than that in the low MAI group (difference: 0.22 ± 0.09; *p* = 0.013).

Based on these results, a multiple logistic regression analysis was conducted (Table 4). A higher number of FTUs significantly reduced the risk of decreased masticatory performance (adjusted odds ratio (OR): 0.692; 95% confidence interval (CI): 0.508–0.942); *p* = 0.019). High tone (adjusted OR: 1.235; 95% CI: 1.057–1.443; *p* = 0.008) and low elasticity (adjusted OR: 5.470; 95% CI: 1.325–22.589; *p* = 0.019) of the masseter muscles significantly increased the risk of decreased masticatory function.

## 4. Discussion

To promote healthy aging, appropriate nutritional intake and normal masticatory function is essential. When the masticatory function is impaired in older adults, dental treatments are primarily considered. However, these treatments are different from community-based integrated oral health management strategies. Therefore, a comprehensive analysis of the factors that affect the masticatory function in community-dwelling older adults is necessary for establishing additional management strategies to effectively improve their masticatory function. In this study, the objective mixing ability test, which is a valid assessment of masticatory function, was performed to analyze the various risk factors of decreased masticatory performance.

The number of remaining teeth, number of FTUs, symptoms of periodontal disease and TMD, and the tension, elasticity, and dynamic stiffness of the masseter muscles were found to be correlated with the MAI (Table 2). Various studies have previously demonstrated the relationship between masticatory function and the number of remaining teeth, number of FTUs, and the symptoms of periodontal disease and TMD [29,30,31]. The present observation that the number of FTUs, which also accounts for the occlusal state of the teeth, had greater effects on masticatory performance than the number of remaining teeth is consistent with that of previous studies [8]. One of the factors with a formidable influence on tooth loss is periodontal disease [32]. In the present study, participants with subjective symptoms of periodontal disease in the last three months before the study had a significantly lower MAI than those without periodontal disease symptoms. Additionally, participants who experienced pain, clicking, or trismus of the temporomandibular joints during the last three months before the study had a significantly lower MAI than those without these symptoms. These results are consistent with those of previous studies that reported that TMD pain can reduce chewing ability [33].

Moreover, tension and elasticity of the masseter muscles were also found to be significantly different between patients with a high MAI and those with a low MAI after adjusting for symptoms related to periodontal disease and TMD, both of which directly affect tooth loss and the opening and closing movements of the lower jaw (Table 3). Therefore, this is the first study to directly compare the relationship between the MAI and the tone and biomechanical properties of the masseter muscles. With aging, muscles throughout the body begin to atrophy, leading to decreased elasticity and increased tension and stiffness [34]. It was observed in the present study that the tension and elasticity of the masseter muscles increased and decreased, respectively, as the masticatory function decreased, suggesting that the atrophy of masseter muscles progresses further in older adults with decreased masticatory performance. Previous studies have shown that the cross-sectional area and the radiographic density of the masseter and medial pterygoid muscles decrease with aging [35], and Murakami et al. [36] reported that a decrease in muscle mass can significantly decrease chewing ability. Therefore, high tension or low elasticity of the masseter muscles may affect the decline in masticatory performance.

It was also found that the number of FTUs and the tension and elasticity of the masseter muscles are risk factors associated with decreased masticatory performance after adjusting for confounding factors (Table 4). Increased tension and decreased elasticity in the masseter muscles were significantly associated with decreased masticatory performance. An increase in the number of FTUs was significantly associated with increased masticatory performance. As chewing ability is closely related to the number and distribution of teeth, the number of FTUs is considered to have a significant impact on chewing ability in older adults [8]; the results of the present study support this notion. In our study, the tension and elasticity of the masseter muscles as well as the number of FTUs were identified as important risk factors that affect masticatory function. One of the most important clinical indicators of masticatory performance is the maximum bite force, which is greatly affected by the cross-sectional area of the masseter muscle [37,38]. The present findings are consistent with those of previous studies that reported that an increase in the tension or a decrease in the elasticity of the masseter muscles can affect the masticatory performance [11,13,37,39].

Older adults with decreased chewing ability consume a relatively soft diet, which may lead to nutritional imbalances and systemic diseases. Han and Kim [40] reported that edentulous patients without dentures had lower potassium, niacin, and vitamin C intake than denture-wearers. Furthermore, the decrease in masticatory function with aging leads to decreased sensory input of the nervous system and cerebral blood flow [5,41], which may result in systemic deteriorations of elderly patients. In a study by Hwang et al. [28], the dynamic balance of elderly participants with low masticatory efficiency was lower than that of elderly participants with high masticatory efficiency, which may lead to an increased fall risk. Bae and Park [42] reported that denture-wearers had decreased neck muscle strength than those without dentures, which may cause the body to bend forward. Moreover, previous studies reported that masticatory function may affect cognitive processing speed [43], physical reaction time [44], and cerebral blood oxygen-dependent signal [45]. Other studies have reported that the restoration of masticatory function can improve arousal and modulate cognitive functions [46]. These findings suggest that management strategies to improve masticatory performance in the elderly should not only seek to restore the dental arch but also to improve the muscle function of the oral and maxillofacial region. In a study by Kim et al. [47], the MAI was significantly increased after only 8 weeks of simple oral exercises; further, Morita et al. [23] reported that masticatory performance is associated with the muscle power of the whole body. Therefore, strengthening the muscle function of the oral and maxillofacial region will improve not only masticatory performance [48] but also the overall physical performance of the elderly [2].

This study has a few limitations. First, the direct causal relationship between factors related to masticatory performance cannot be elucidated as this was a cross-sectional study. Additionally, the participants of this study were selected via convenience sampling from a specific community in South Korea; therefore, the results cannot be generalized. However, to overcome these limitations, this study was performed after calculating the sample size needed for the study design. Second, the two questionnaires used in this study for assessing periodontal disease and TMD symptoms had not been previously validated. Nonetheless, the questionnaires consisted of items that assessed the most critical aspects related to the clinical symptoms of periodontal disease and TMD. Third, objective measurements of the masticatory function in older adults were obtained using the mixing ability test. The two-colored wax cubes used in this study may have been difficult to chew for the participants, which could account for the lower MAI in this study than that reported in a previous study [26]. Bias in the data was minimized as much as possible via standardization and the performance of an equal number of masticatory movements on the right and left sides. Fourth, although several muscles in the oral and maxillofacial region are involved in mastication, the tone and biological properties of only the masseter muscles were measured. Finally, periodontal disease and TMD-related symptoms were investigated in this study using a self-reported questionnaire. To minimize possible response errors, sufficient explanation was provided to the participants about the intent of the questions. Therefore, future studies should consider an increased sample size and stratified research design to compensate for the limitations described earlier, and they also should consider methods to clinically evaluate the symptoms of periodontal disease and TMD. Furthermore, the factors affecting mastication in older adults should be determined by observing the muscles related to dysphagia in addition to the masticatory muscles. Nevertheless, this is the first study to suggest that MAI, an objective indicator of masticatory function, can be affected by the tension and elasticity of the masseter muscles. This study comprehensively and objectively analyzed the effects of several factors on masticatory performance by analyzing the characteristics of the masseter muscle and FTUs. Therefore, the present findings can be used to establish integrated whole-body health management strategies for community-dwelling older adults.

## 5. Conclusions

The number of FTUs and the tension and elasticity of the masseter muscles are associated with MAI. Training strategies that can improve muscle function and prosthetic treatments can be used to improve the masticatory ability of older adults. These therapies serve to efficiently manage the oral health as well as the overall health of older adults.

## Figures and Tables

**Table 1:** Oral and maxillofacial characteristics of the participants according to mixing ability index.

Variable	Total	Mixing Ability Index		
		≥51.86 (*n* = 40)	<51.86 (*n* = 40)	*p*-Value
Age	75.10 ± 5.64	74.95 ± 5.49	75.25 ± 5.85	0.814 *
Sex				
Male	35 (43.8)	14 (35.0)	21 (52.5)	0.176 †
Female	45 (56.3)	26 (65.0)	19 (47.5)	
Mixing ability index	55.22 ± 19.59	70.49 ± 13.65	39.95 ± 10.60	<0.001 *
Number of remaining teeth	18.60 ± 4.38	19.45 ± 4.33	17.75 ± 4.33	0.083 *
Number of functional tooth units	7.29 ± 1.88	7.70 ± 1.60	6.89 ± 2.06	0.052 ^‡^
Symptoms related to periodontal disease	1.66 ± 1.08	1.45 ± 1.03	1.88 ± 1.09	0.078 ^‡^
Symptoms related to temporomandibular disease (TMD)	0.74 ± 0.87	0.58 ± 0.71	0.90 ± 0.98	0.095 ^‡^
Salivary secretion rate (mL/min)	1.62 ± 0.62	1.66 ± 0.69	1.58 ± 0.54	0.564 ^†^
Oscillation frequency of masseter (Tension, Hz)	19.03 ± 3.67	17.83 ± 3.21	20.23 ± 3.75	0.003 *
Logarithmic decrement of masseter (Elasticity)	1.49 ± 0.38	1.39 ± 0.41	1.60 ± 0.32	0.011 *
Dynamic stiffness of masseter (N/m)	321.41 ± 49.12	314.05 ± 43.14	328.76 ± 54.00	0.182 *

All values are presented as mean ± standard deviation or *n* (%). * Independent *t*-test; ^†^ Chi-square test; ^‡^ Mann–Whitney U test.

**Table 2:** Pearson’s correlation coefficient for the association with mixing ability index.

Variables	MAI	
	*r*	*p*-Value
Age	0.024	0.834
Number of remaining teeth	0.270	0.015 *
Number of functional tooth units	0.404	<0.001 **
Symptoms related to periodontal disease	−0.307	0.006 **
Symptoms related to temporomandibular disease	−0.283	0.011 *
Stimulated salivary secretion rate (mL/min)	0.015	0.897
Oscillation frequency of masseter (Tension, Hz)	−0.357	0.001 **
Logarithmic decrement of masseter (Elasticity)	−0.317	0.004 **
Dynamic stiffness of masseter (N/m)	−0.371	0.001 **

Data are presented as *r* or *p*-value. MAI, mixing ability index. Pearson’s correlation analysis, * *p* < 0.05, ** *p* < 0.01.

**Table 3:** Comparison of tone and biomechanical properties of the masseter muscles according to the mixing ability index.

Variables	*n*	Oscillation Frequency (Tension, Hz)	*p*-Value	Logarithmic Decrement (Elasticity)	*p*-Value	Dynamic Stiffness (N/m)	*p*-Value
MAI							
≥51.86	40	17.83 ± 3.21	0.006 ^*^	1.39 ± 0.41	0.013 *	314.05 ± 43.14	0.389
<51.86	40	20.23 ± 3.75		1.60 ± 0.32		328.76 ± 54.00	

Data are presented as mean ± standard deviation. MAI, mixing ability index. * *p*-value is adjusted via analysis of covariance for number of functional tooth units, symptoms related to periodontal disease, and symptoms related to temporomandibular disease, α = 0.05.

**Table 4:** Factors that decrease mixing ability index.

Predictor Variables	Model I (Crude)			Model II (Adjusted)		
	OR	95% CI	*p*-Value	OR	95% CI	*p*-Value
Number of functional tooth units	0.702	0.506–0.974	0.034 ^†^	0.692	0.508–0.942	0.019 ^‡^
Oscillation frequency of the masseter (Tension, Hz)	1.225	1.045–1.435	0.012 ^†^	1.235	1.057–1.443	0.008 ^‡^
Logarithmic decrement of the masseter (Elasticity)	5.161	1.227–21.700	0.025 ^†^	5.470	1.325–22.589	0.019 ^‡^
Symptoms related to periodontal disease	1.077	0.654–1.776	0.770	-	-	-
Symptoms related to temporomandibular disease	1.424	0.784–2.584	0.245	-	-	-

OR, Odds ratio; CI, confidence intervals. ^†^ *p*-values obtained from logistic regression analysis with all possible variables entered at α = 0.05. ^‡^ *p*-values obtained from a multivariable analysis of logistic regression with backward elimination at α = 0.05.

## Data Availability

The data presented in this study are available on request from the corresponding author.

## Supplementary Materials

The following are available online at https://www.mdpi.com/article/10.3390/ijerph18084286/s1, File S1: Questionnaire on periodontal disease; File S2: Questionnaire on temporomandibular disease.
