# Supplementary material for: Influential Factors of Masticatory Performance in Older Adults: A Cross-Sectional Study
Source: Int J Environ Res Public Health. 2021 Apr 18;18(8):4286. doi: 10.3390/ijerph18084286 (PMC8072885; doi:10.3390/ijerph18084286)
Supplement: Supplementary file 1 [file ijerph-18-04286-s001.zip › ijerph-1186156-supplementary.pdf]

File S1: Questionnaire on periodontal disease

1) Do your gums bleed sometimes? ① Yes ② No

2) Do your gums swell? ① Yes ② No

3) Do you sometimes feel like your teeth are rising? ① Yes ② No

4) Do you have any shaky teeth? ① Yes ② No

File S2: Questionnaire on temporomandibular disease

1) Do you feel pain in the jaw when you chew or open your mouth wide? ① Yes ② No

2) Does your jaw make a certain sound when you open your mouth? ① Yes ② No

3) Is it hard to open your mouth widely? ① Yes ② No
